# Optimal Current Transfer in Dendrites

**DOI:** 10.1371/journal.pcbi.1004897

**Published:** 2016-05-04

**Authors:** Alex D. Bird, Hermann Cuntz

**Affiliations:** 1 Warwick Systems Biology Centre, University of Warwick, Coventry, United Kingdom; 2 Warwick Systems Biology Doctoral Training Centre, University of Warwick, Coventry, United Kingdom; 3 School of Life Sciences, University of Warwick, Coventry, United Kingdom; 4 Ernst Strüngmann Institute (ESI) for Neuroscience in Cooperation with Max Planck Society, Frankfurt-am-Main, Germany; 5 Frankfurt Institute for Advanced Studies, Frankfurt-am-Main, Germany; University of Edinburgh, UNITED KINGDOM

## Abstract

Integration of synaptic currents across an extensive dendritic tree is a prerequisite for computation in the brain. Dendritic tapering away from the soma has been suggested to both equalise contributions from synapses at different locations and maximise the current transfer to the soma. To find out how this is achieved precisely, an analytical solution for the current transfer in dendrites with arbitrary taper is required. We derive here an asymptotic approximation that accurately matches results from numerical simulations. From this we then determine the diameter profile that maximises the current transfer to the soma. We find a simple quadratic form that matches diameters obtained experimentally, indicating a fundamental architectural principle of the brain that links dendritic diameters to signal transmission.

## Author Summary

Neurons take a great variety of shapes that allow them to perform their different computational roles across the brain. The most distinctive visible feature of many neurons is the extensively branched network of cable-like projections that make up their dendritic tree. A neuron receives current-inducing synaptic contacts from other cells across its dendritic tree. As in the case of botanical trees, dendritic trees are strongly tapered towards their tips. This tapering has previously been shown to offer a number of advantages over a constant width, both in terms of reduced energy requirements and the robust integration of inputs at different locations. However, in order to predict the computations that neurons perform, analytical solutions for the flow of input currents tend to assume constant dendritic diameters. Here we introduce an asymptotic approximation that accurately models the current transfer in dendritic trees with arbitrary, continuously changing, diameters. When we then determine the diameter profiles that maximise current transfer towards the cell body we find diameters similar to those observed in real neurons. We conclude that the tapering in dendritic trees to optimise signal transmission is a fundamental architectural principle of the brain.

## Introduction

Integration of synaptic inputs relies on the propagation of currents arising from sources across the dendritic tree. Whilst active processes strongly contribute to current flow in most neurons [1–3], understanding the passive backbone to transmission is key to an intuitive grasp of dendritic function; the results of Wilfrid Rall in highlighting the properties of cylindrical dendrites [4–6] are of foundational importance in compartmental modelling and computational neuroscience. Dendrites are, however, not generally cylindrical. The distal taper seen in the majority of all cases appears to both increase passive current flow towards the soma [7–9], thus reducing the energy requirements of active compensatory processes, and to contribute to the phenomenon of dendritic democracy, where somatic voltage amplitudes are equalised between different synaptic sites [10–12].

Common numerical approaches to modelling taper treat a dendritic cable as a series of cylinders or linearly tapering frusta [5,13–18]. Whilst these techniques are accurate and powerful, there is much to be gained from an analytical solution to the voltage in terms of intuition and computational speed. A number of solutions for the voltage in non-uniform cables exist [19–21], but these involve either the more tractable cases of varying electrotonic properties with constant radius or are limited to a few forms of radius taper.

We present an asymptotic approximation to the voltage in dendrites with any given taper profile using the insight that voltage attenuation is substantially faster than radius change in realistic morphologies. A particularly appealing prospect for such an approach is that the optimal taper profile to transfer distal synaptic currents to the soma can then be derived using variational calculus. The optimal taper profile is shown to match the results of numerical optimisation and predict radii measured experimentally from a number of different cell classes.

## Results

### Accurate approximation of voltage in a cable with arbitrary radius profile

A length of passive dendrite tapers with radius at distance *x* given by *r*(*x*). The leak conductance per unit area is denoted *g*_*l*_, the axial resistance *r*_*a*_, and the membrane time constant *τ*. Then the voltage above equilibrium *v*(*x*, *t*) at location *x* and time *t* obeys the generalised cable equation

$$\tau \frac{\partial v}{\partial t}=-v+\frac{1}{2r_{a}g_{l}r\left(x\right)}\frac{1}{\sqrt{1+{\left(r^{\prime }\left(x\right)\right)}^{2}}}\frac{\partial }{\partial x}\left[r^{2}\left(x\right)\frac{\partial v}{\partial x}\right]$$ (1)

The rate of voltage attenuation is generally significantly steeper than the rate of change of dendritic radius, allowing use of the method of multiple scales [22] to accurately approximate the voltage evolution. We introduce *X* = *ϵx* as the ‘slow’ taper variable and treat it as independent of *x*. Large regions of most dendritic trees admit small values of *ϵ* (~0.01, S1 Fig).

Expanding in *ϵ*, gives the first-order steady-state solution (see Methods)

$$v\left(x\right)=\sqrt{\lambda \left(x\right)}\left[Ae^{\int_{x^{\prime }}^{x}\frac{1}{\lambda \left(s\right)}ds}+Be^{-\int_{x^{\prime }}^{x}\frac{1}{\lambda \left(s\right)}ds}\right]$$ (2)

for $\lambda (x)\;=\;\sqrt{\frac{r(x)}{2r_{a}g_{l}}}$ the location-dependent electrotonic length, *x′* a site of current injection, and constants *A* and *B* determined by the boundary constraints.

**Fig 1:**
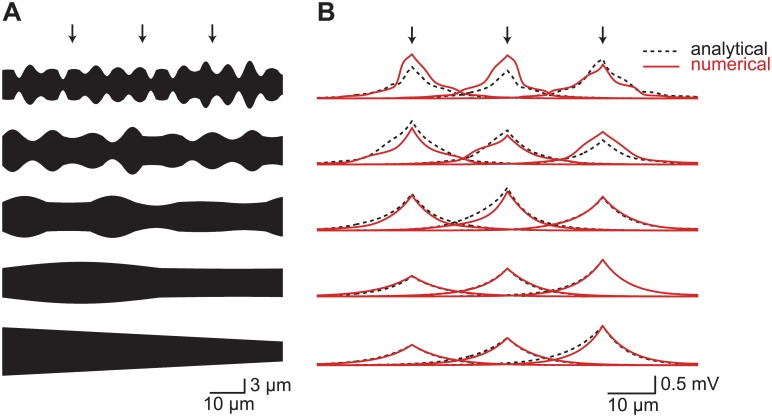
Cable theory with arbitrary diameters—accuracy of the first-order analytical approximation. (**A**) Sample radius profiles illustrating the accuracy of the analytical approximation. The radius profiles are smoothed from top to bottom by increasing the period and reducing the amplitude of the radius change. Current is injected separately at the three points indicated by arrows. (**B**) Comparison of numerical (red solid lines) and analytical (black dashed lines) voltages in dendrites with varying radius profiles. As the radius changes more slowly, the first-order approximation becomes more accurate (from top to bottom). Note that the large diameters used in this figure emphasize the difference between the numerical and analytical solution. Using smaller diameters as are usual everywhere but at the most proximal dendrites, the analytical approximation becomes essentially a perfect match.

To demonstrate the validity of this approximation, we generated a series of artificial dendritic cables and compared the first-order approximation to the numerical solution (Fig 1). The artificial cables have periodically changing diameters with a random amplitude for each period. Increasing the period and reducing the amplitude smooths the artificial cable, reducing *ϵ* and improving the approximation. The multiple-scales solution provides an accurate approximation to the voltage in realistic dendritic cables.

The simple form seen here allows for the usual features of cable theory to be reconstructed. In particular, standard analytic results for voltage propagation in complex dendritic structures and time-dependence have easy analogies in tapering cables. Greater accuracy can also be achieved, up to a point, by taking higher-order terms in *ϵ*. These results are shown in the Supporting Information.

### Optimal taper for a single dendritic cable

An analytical expression for the voltage at leading order allows for study of the optimal dendritic radius profile to propagate synaptic currents towards the soma. Previous work in this direction lacked a continuous representation of the voltage profile and used numerical methods to explore optimality [9]. Calculus of variations provides a framework in which to define the optimal profile (for the leading-order component of the voltage) continuously.

Given a dendritic cable of length *L* with volume *V* and distal (minimal) radius *r*_*L*_, the goal is to maximise the voltage at the proximal end of a dendritic cable for synaptic currents arising at all points along the cable. This means maximising the functional

J=∫0L1λ72(x′)e−∫0x′1λ(s)dsdx′ (3)

where the effect of ‘reflected’ current at the distal end has been neglected due to the relatively fast time course of excitatory potentials.

**Fig 2:**
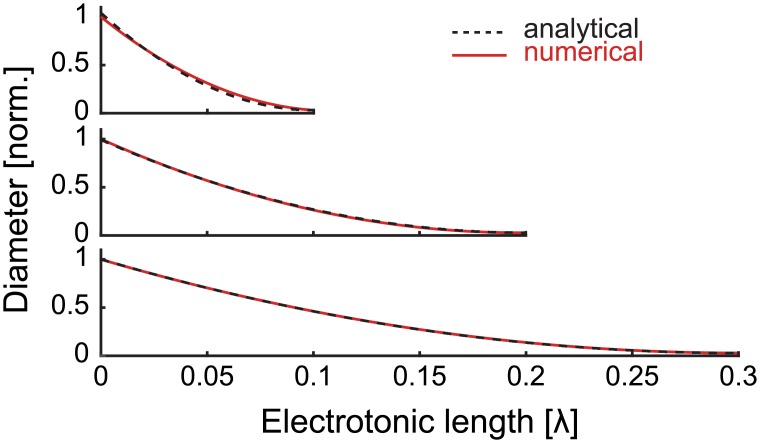
Diameter profiles to optimise current transfer. Comparison of non-parametrically optimised (red solid lines) and theoretical (black dashed lines) radius profiles for different electrotonic lengths of dendritic branch. The theoretical profile tapers more strongly in the shortest case as it neglects the increase in distal input resistance from the sealed end. The scaling parameters *α* corresponding to Eq 4 are 101.1, 24.08, and 10.81 respectively.

The maximisation gives an optimal radius profile of (see Methods)

$$r\left(x\right)=\alpha {\left(L-x\right)}^{2}+r_{L}$$ (4)

where *α* is fitted to match the volume of the cable *V*. This profile matches the results of numerical optimisation (Fig 2).

### Optimal taper in a dendritic tree

Having found the optimal single cable for voltage propagation, it remains to be shown how far real dendritic trees correspond to this optimality. Wilfrid Rall [4] showed that if the diameters of cylindrical sections at dendritic branch points satisfied the relationship $d_{p}^{3/2}\;=\;d_{c1}^{3/2}+d_{c2}^{3/2}$, matching the conductance across the branch, then the entire dendritic tree could be collapsed to a single cylinder. Rall’s relationship is rarely satisfied in real dendrites [20,23,24]. Using a Rallian diameter ratio at a branch, however, allows us to ensure that the transition between parent and daughter branches obeys the quadratic optimality condition. This makes it possible to map quadratic radii onto complex dendritic morphologies by constraining dendrites to locally obey optimality (see Methods). The resulting predicted morphologies show how far dendritic trees are globally optimised to transmit and equalise current transfer.

**Fig 3:**
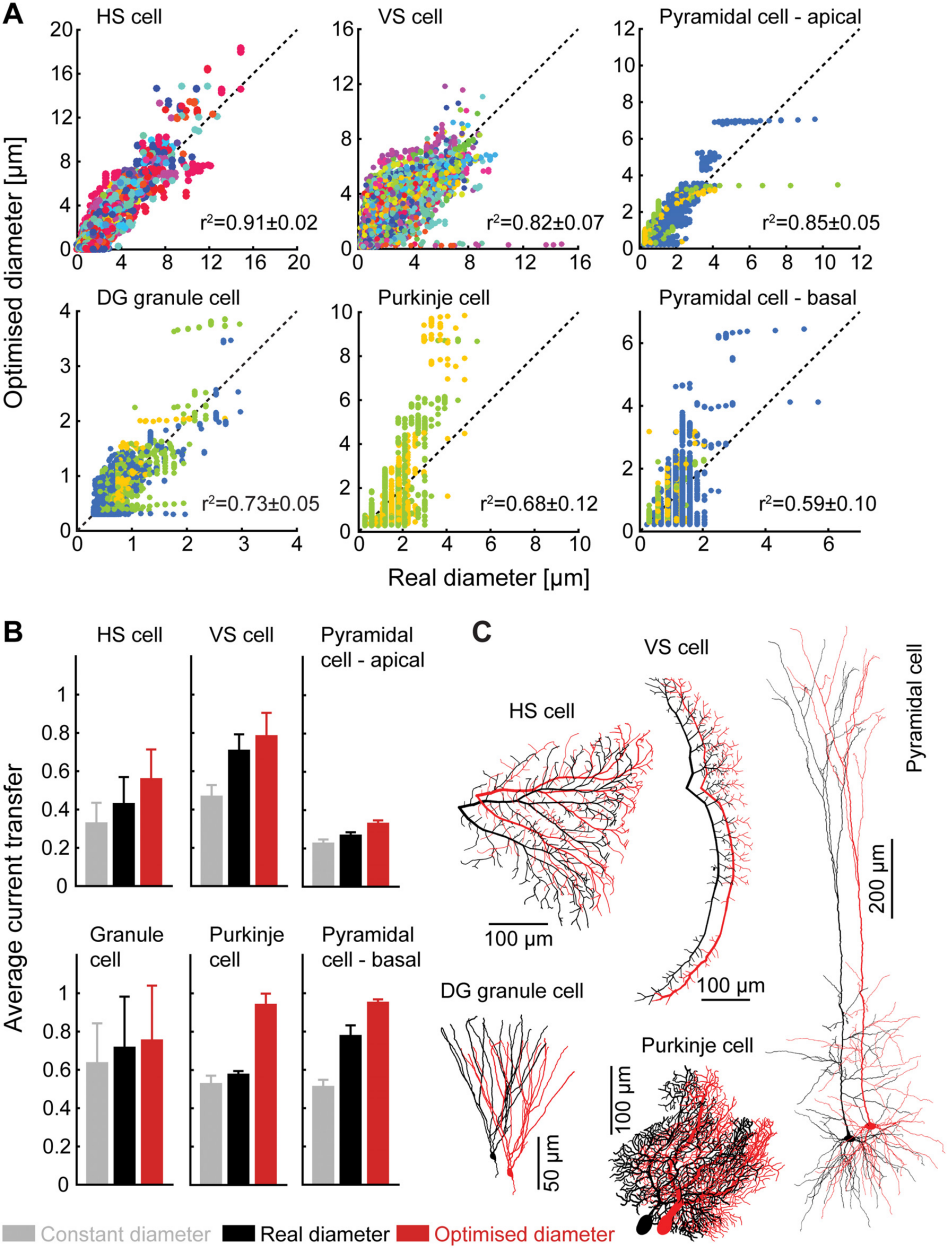
Real dendrites are constrained by current transfer optimality. (**A**) Scatter plots of measured radius against optimal radius and correlation coefficients for different cell classes. Different colours denote different cells of the same class. (**B**) Average current transfer to the root from different cell classes with (from left to right) constant, measured, and optimal radii. (**C**) Examples of reconstructed sample morphologies and the same morphologies with optimal diameter profiles. The neurons shown in this figure are two types of fly neurons (HS cell and VS cell with specific membrane conductance of *g*_*l*_ = 5 × 10^−4^ S/cm^2^) and three mammalian neurons (dentate gyrus granule cells with *g*_*l*_ = 4 × 10^−5^ S/cm^2^, and cerebellar Purkinje cells and neocortical Layer V neurons with *g*_*l*_ = 5 × 10^−5^ S/cm^2^).

We have selected a number of neuronal classes with a broad array of functions to examine the validity of our predictions (Fig 3A). It should be noted here that obtaining reliable measurements of dendritic radius is experimentally very challenging and this makes exact comparisons difficult. Different cell types satisfy the equivalent quadratic criterion to different degrees. Of the cell classes studied, the best agreement was for fly neurons, which might be considered genetically more hardwired [25,26]. In terms of mammalian neurons, the best agreement was found for dentate gyrus granule cells. These cells are known to both obey Wilfrid Rall’s branching criterion [27] and undergo continuous replacement throughout life [28]. These results suggest that our model might best match cells with a stereotypic morphology and therefore an initially optimal passive backbone.

The diameter profiles of apical and basal dendrites in cortical pyramidal cells match optimality to different degrees. The apical tree appears well described in terms of quadratic equivalent taper, despite differences at the trunk of the apical dendrite. As the apical dendrite might be more strongly specialised in propagating dendritic spikes, deviations might not be surprising. The predicted diameter profile for the basal dendrites was less accurate. Here there appear to be sections of the reconstruction that are much more voluminous than their length relative to other branches would suggest. This might imply that the relationship between nearby cells exerts a stronger influence than is seen elsewhere and that local cortical microcircuits display preferential connections in some directions.

No agreement was found for cerebellar Purkinje cells, where the general taper profile is much shallower than would be expected and dendrites often exhibit alternate bulges and narrower regions. The distinctive layered structure of the cerebellum means that excitatory synaptic inputs arrive in distinct locations, strong synapses from climbing fibres proximally and individually weaker, but much more numerous, synapses from parallel fibres distally. These two types of inputs are implicated in different spiking patterns, complex and simple spikes respectively, and the functional relationship between the two is beyond the scope of our general optimality principle.

Structurally, the agreement between ideal and observed morphologies therefore varies with specific function, but the model provides a good fit to large regions of many dendritic trees. We can, however, show how well the quadratic taper performs for all classes studied (Fig 3B). Plotting the current transfer from all nodes to the soma illustrates the advantages of quadratic taper against a constant diameter across the tree and provides a slight advantage over observed morphologies. Our results highlight the importance of a specific form of taper in maximising current transfer and equalising synaptic inputs.

Interestingly, for the dendrites where current transfer loss was largest because of either the size (the apical dendrite of the pyramidal cell) or because of a high membrane conductivity (as was the case in the fly neurons), the diameters tended to be better predicted by optimal current transfer. Where cells deviate substantially from passive optimality, for example specifically along the trunk of the apical dendrite of a pyramidal cell or across a Purkinje cell, there is evidence that these sections of dendrite favour functions other than the unidirectional propagation of excitatory synaptic currents towards the soma.

## Discussion

The fact that voltages in dendrites typically decay much more quickly than radii allows us to make a simple and accurate approximation to the propagation of currents across real dendritic trees. The compact form of the voltage approximation allows for a straightforward reproduction of the standard results of cable theory [4–6]. Further, this result allows the continuous optimum taper profile for transmitting synaptic currents to the soma to be deduced. The optimal radius profile tallies with notions of both dendritic democracy [11,12,29] and energy optimisation [9] and provides a close match to reconstructed dendritic morphologies across a range of cell classes.

Dendrites perform an array of non-linear computations involving active processes and local inhibition; the general principle of global passive optimality does not explain every facet of dendritic function, but does provide an important new intuition. The simple forms of both voltage and optimal radius link signal transmission and dendritic diameters, allowing a clearer intuitive understanding of the function of dendritic trees.

## Methods

### First-order multiple scales approximation

Consider the homogenous steady-state voltage equation for a cable with arbitrary radius *r*(*x*)

$$\frac{\partial }{\partial x}\left[r^{2}\left(x\right)\frac{\partial v}{\partial x}\right]-2r_{a}g_{l}r\left(x\right)\sqrt{1+{\left(r^{\prime }\left(x\right)\right)}^{2}}v=0$$ (5)

with boundary conditions

$$\begin{array}{cc} {\frac{dv}{dx}|}_{x=L}=0 & \;\;\;\;\;\;\;\;\;\;\;\;\;\;\;\;\;\;\;\;\;\;\;\;\underset{x\to -\infty }{\text{lim}}v=0 \end{array}$$ (6)

*r* typically changes more slowly as a function of *x* than *v* does, specifically *r*(*x*) = *ρ*(*ϵx*) for *ϵ* ≪ 1. S1 Fig shows typical values of *ϵ* for a range of reconstructed morphologies. It is possible to treat the ‘fast' voltage length variable *x* and the ‘slow' radius length variable *ϵx* as independent using the method of multiple scales. Then $\frac{dr}{dx}\;=\;\epsilon \frac{d\rho }{dx}$ and the steady-state voltage equation becomes

$$0=\rho^{2}\frac{d^{2}v}{dx^{2}}+2\epsilon \rho \rho \prime \frac{dv}{dx}-2r_{a}g_{l}\rho \sqrt{1+{\left(\epsilon \rho \prime \right)}^{2}}v$$ (7)

Introducing the new variable *w* such that *w* = *ρ*^*ϵ*^*v* allows us to write the voltage equation as

$$\begin{array}{l} 0=\frac{d^{2}w}{dx^{2}}-\left(2r_{a}g_{l}\frac{\sqrt{1+{\left(\epsilon \rho \prime \right)}^{2}}}{\rho }+\frac{\epsilon^{2}\left(\rho^{2}\rho^{\prime \prime \prime }-3\rho \rho^{\prime }\rho^{\prime \prime }+2{\left(\rho \prime \right)}^{3}\right)}{2\rho^{3}}+\frac{{\left(\epsilon \rho \prime \right)}^{2}}{4\rho^{2}}\right)w\\ 0=\frac{d^{2}w}{dx^{2}}-f\left(\epsilon x\right)w \end{array}$$ (8)

Note that $\sqrt{1+(\epsilon \rho '{)}^{2}}\approx 1+\frac{{\left(\epsilon \rho '\right)}^{2}}{2}$ and that −*f*, the coefficient of *w*, will always be negative making the solution appropriately non-oscillatory. We seek solutions of the form

$$w\left(x\right)=\mu \left(\epsilon x\right)e^{\int_{}^{x}\sigma \left(\epsilon s\right)ds}$$ (9)

for *μ* and *σ* real. Substituting this into the above equation gives at first order

$$w\left(x\right)=\frac{\rho {\left(x\right)}^{1/4}}{{\left(2r_{a}g_{l}\right)}^{1/4}}\left[Ae^{\int_{}^{x}\sqrt{\frac{2r_{a}g_{l}}{\rho \left(s\right)}}ds}+Be^{-\int_{}^{x}\sqrt{\frac{2r_{a}g_{l}}{\rho \left(s\right)}}ds}\right]$$ (10)

for some constants *A* and *B*. Writing $\lambda (x)\;=\;\sqrt{\frac{\rho (x)}{2r_{a}g_{l}}}$ as the distance-dependent electrotonic length gives the leading-order form

$$v\left(x\right)\approx \sqrt{\lambda \left(x\right)}\left[Ae^{\int_{}^{x}\frac{1}{\lambda \left(s\right)}ds}+Be^{-\int_{}^{x}\frac{1}{\lambda \left(s\right)}ds}\right]$$ (11)

### Current injection

To determine the response to a current injection of magnitude *I*_*app*_ at site *x′*, note that the Green's function *g*(*x*, *x′*) solves the equation

$$\frac{\partial }{\partial x}\left[r^{2}\left(x\right)\frac{\partial g}{\partial x}\right]-2r_{a}g_{l}r\left(x\right)\sqrt{1+{\left(r^{\prime }\left(x\right)\right)}^{2}}g=\delta \left(x-x\prime \right)$$ (12)

subject to a given set of boundary conditions. Away from *x′*, the solution is given by the homogenous voltage above, namely for *x* < *x′*

$$g_{x<x\prime }\left(x,x\prime \right)=\sqrt{\lambda \left(x\right)}B_{1}e^{-\int_{}^{x}\frac{1}{\lambda \left(s\right)}ds}$$ (13)

using the fact that voltages are required to decay towards the soma. For *x* > *x′*

$$g_{x>x\prime }\left(x\right)=\sqrt{\lambda \left(x\right)}\left[A_{2}e^{\int_{}^{x}\frac{1}{\lambda \left(s\right)}ds}+B_{2}e^{-\int_{}^{x}\frac{1}{\lambda \left(s\right)}ds}\right]$$ (14)

Here, the sealed-end condition gives the relationship between the constants as

$$B_{2}=A_{2}\left(\frac{2+\lambda \prime \left(L\right)}{2-\lambda \prime \left(L\right)}\right)e^{2\int_{x\prime }^{L}\frac{1}{\lambda \left(s\right)}ds}$$ (15)

Continuity of voltage at *x′* ensures

$$B_{1}=A_{2}\left(1+k\right)$$ (16)

for *k* the ratio between *A*_2_ and *B*_2_ given by the sealed end condition. Conservation of current at the point of injection relates all three constants

$$\begin{array}{c} {g^{\prime }}_{x<x^{\prime }}\left(x^{\prime }\right)+\frac{r_{a}}{\pi \rho^{2}\left(x\prime \right)}I_{app}={g^{\prime }}_{x<x^{\prime }}\left(x^{\prime }\right)\\ B_{1}\left(\lambda^{\prime }\left(x^{\prime }\right)-2\right)+\frac{2r_{a}\sqrt{\lambda \left(x^{\prime }\right)}}{\pi \rho^{2}\left(x\prime \right)}=A_{2}(\lambda^{\prime }\left(x^{\prime }\right)+2+k(\lambda^{\prime }\left(x^{\prime }\right)-2))) \end{array}$$ (17)

giving the coefficients in terms of the initial parameters as

$$\begin{array}{l} B_{1}=\frac{r_{a}\sqrt{\lambda \left(x\prime \right)}}{2\pi \rho^{2}\left(x^{\prime }\right)}\left[1+\left(\frac{2-\lambda \prime \left(L\right)}{2+\lambda \prime \left(L\right)}\right)e^{-2\int_{x\prime }^{L}\frac{1}{\lambda \left(s\right)}ds}\right]I_{app}\\ A_{2}=\frac{r_{a}\sqrt{\lambda \left(x\prime \right)}}{2\pi \rho^{2}\left(x^{\prime }\right)}\left[\frac{2-\lambda \prime \left(L\right)}{2+\lambda \prime \left(L\right)}\right]e^{-2\int_{x\prime }^{L}\frac{1}{\lambda \left(s\right)}ds}I_{app}\\ B_{2}=\frac{r_{a}\sqrt{\lambda \left(x\prime \right)}}{2\pi \rho^{2}\left(x^{\prime }\right)}I_{app} \end{array}$$ (18)

Note that *B*_1_(*x′*) is the input resistance at site *x′*.

As we are primarily interested in voltage at the proximal terminal of the dendrite, we focus on the solution in the region *x* < *x′* and evaluate the voltage at *x* = 0. The first-order approximation holds for a region of size *ϵ*^−1^ away from the site of current injection. Section 4 of the S1 Text describes how to extend this approximation to account for higher-order terms, which can allow for greater accuracy (S2 Fig), as well as voltage transients and voltage propagation in branched structures (S3 Fig).

### Optimality of current transfer

It is possible to use calculus of variations to study the functions *r*(*x*) that give extremal values of a functional *J*[*x*, *r*, *r′*]. We seek to define the radius profile that maximises current transfer. In this case we seek to maximise the total current transfer to the proximal end *x* = 0, from all injection sites *x′* = 0 to *x′* = *L*, under constraints of fixed terminal radii or total cable volume. Writing the voltage at 0 due to current injection at *x′* as *v*(0, *x′*) such that

$$v\left(0,x\prime \right)=\frac{r_{a}\sqrt{\lambda \left(x\prime \right)}}{2\pi \rho^{2}\left(x\prime \right)}\left[1+\left(\frac{2-\lambda \prime \left(L\right)}{2+\lambda \prime \left(L\right)}\right)e^{-2\int_{x\prime }^{L}\frac{1}{\lambda \left(s\right)}ds}\right]I_{app}\sqrt{\lambda \left(0\right)}e^{-\int_{0}^{x\prime }\frac{1}{\lambda \left(s\right)}ds}$$ (19)

We seek to maximise the functional

$$J=\int_{0}^{L}v\left(0,x\prime \right)dx^{\prime }=\int_{0}^{L}Kdx^{\prime }$$ (20)

*J* is a functional of the functions *λ*(*x*) and ∫x1λ(s)ds. It is convenient to write Λ(x) = ∫x1λ(s)ds so that Λ'(x) = 1λ(x). For *J* to take a maximal or minimal value, it is necessary for the integrand *K* to satisfy the Euler-Lagrange equation

$$0=\frac{\partial K}{\partial \Lambda }-\frac{d}{dx}\frac{\partial K}{\partial \Lambda \prime }$$ (21)

with boundary conditions following from the original constraints. Introducing the constants $C_{1}\;=\;\frac{r_{a}\sqrt{\lambda (0)}}{2\pi {\left(2r_{a}g_{l}\right)}^{2}}$ and $C_{2}\;=\;\frac{2-\lambda '(L)}{2+\lambda '(L)}e^{-2\int_{0}^{L}\frac{1}{\lambda (s)}ds}$ allows us to write

K[Λ(x),Λ′(x)]=C1Λ′(x)72[e−Λ(x)+C2eΛ(x)] (22)

The Euler-Lagrange equations give that *J* will not be maximised unless Λ satisfies

$$0=\frac{9}{7}\left[C_{2}e^{\Lambda \left(x\right)}-e^{-\Lambda \left(x\right)}\right]-\frac{9}{2}\frac{\Lambda \prime \prime \left(x\right)}{{\left(\Lambda \prime \left(x\right)\right)}^{2}}\left[C_{2}e^{\Lambda \left(x\right)}+e^{-\Lambda \left(x\right)}\right]$$ (23)

To solve this in terms of elementary functions we introduce a further assumption that current is injected sufficiently far from the distal end for the contribution of ‘reflected' current to the input resistance to be negligible (this applies more generally when considering responses to transient current injection). This assumption is equivalent to making *C*_2_e^Λ(*x*)^ vanishingly small, giving the equation

$$\begin{array}{r} \;\;\;\;\;\;\;\frac{\Lambda^{\prime \prime }\left(x\right)}{{\left(\Lambda^{\prime }\left(x\right)\right)}^{2}}=-\frac{2}{7}\\ \frac{d}{dx}\left[-\frac{1}{\Lambda \prime \left(x\right)}\right]=-\frac{2}{7} \end{array}$$ (24)

Using the definitions of Λ(*x*) and *λ*(*x*), and the boundary conditions gives (for a constant *C*_3_)

$$\begin{array}{ll} \sqrt{\frac{r\left(x\right)}{2r_{a}g_{l}}} & =\pm \frac{2}{7}x+C_{3}\\ r\left(x\right) & =\alpha {\left(L-x\right)}^{2}+r_{L} \end{array}$$ (25)

where *r*_*L*_ is the distal (minimal) radius and *α* is determined by matching volumes or proximal radii as required.

It should be noted that whilst the current transfer functional described here is one of a number of possible functionals to optimise, it provides a straightforward and robust description of dendritic function. Further, with temporally active conductance-based synapses, there will be a potential further attenuation of more distal inputs that is beyond the scope of this study.

### Algorithm for constructing an optimal equivalent cable

The final comparison of optimal dendritic taper to real morphologies requires an algorithm for mapping a quadratic taper onto complex branched structures. In particular it requires a principled consideration of the way to distribute dendritic radius at branch points. We seek to equalise conductance at branch points using Rall's 3/2 power relationship; that for a parent radius *r*_0_, and daughter radii *r*_1_ and *r*_2_, then $r_{0}^{3/2}\;=\;r_{1}^{3/2}+r_{2}^{3/2}$. The ratio between *r*_1_ and *r*_2_ is defined by the lengths *l*_1_ and *l*_2_ of the two daughter branches such that $r_{1}/l_{1}^{3/2}\;=\;r_{2}/l_{2}^{3/2}$. The two daughter branches appear to the parent branch to be a single branch with length $l_{0}\;=\;{\left(l_{1}^{3/2}+l_{2}^{3/2}\right)}^{2/3}$. The algorithm for applying these principles to a real dendritic morphology with complex branching structure is described below.

#### i. Obtaining apparent lengths

Starting at the distal termination points of the tree, path lengths are found to the most distal branch points. The ‘apparent length' distal to these branch points is calculated and the process is repeated for every branch point heading towards the root of the tree. This gives an ‘apparent length' for the entire tree and for the daughter branches at each branch point.

#### ii. Distributing radii

The initial radius taper is defined by Eq 4 with *L* given by the apparent length, *r*_*L*_ by the minimal dendritic radius anywhere on the tree and an initial estimate of the proximal radius from the measured physiological maximum. At every branch point the parent radius *r*_0_ is already defined by construction and daughter radii *r*_1_ and *r*_2_ are determined using the ‘apparent lengths' into each branch. This is continued until radii are assigned everywhere on the tree.

#### iii. Matching volumes

This procedure may produce a predicted tree with volume higher or lower than the original morphology. The proximal radius is scaled down or up and step *ii* is repeated until the volumes are matched and an optimal tree with identical volume is found.

### Dendritic morphologies and passive parameters

Five cell classes are discussed in the paper, covering an array of functions and species. All morphologies are publicly available. Blowfly *calliphora vicina* HS (25 examples) and VS (30 examples) neuron morphologies are published with the TREES toolbox [18]. The passive parameters used are axial resistance *r*_*a*_ = 60Ωcm and membrane conductance *g*_*l*_ = 5 × 10^−4^S cm^−2^ for both. Mouse dentate gyrus granule cells (3 examples) are published on ModelDB (Accession no. 95960)[30]. The passive parameters used are *r*_*a*_ = 210Ωcm and *g*_*l*_ = 4 × 10^−5^S cm^−2^. Rat Purkinje cells (2 examples) are published on NeuroMorpho (IDs NMO_00891 and NMO_00892)[31], with *r*_*a*_ = 150Ωcm and *g*_*l*_ = 5 × 10^−5^S cm^−2^. Rat Layer V pyramidal cells (3 examples) are published on ModelDB (Accession no. 139653)[32], with *r*_*a*_ = 150Ωcm and *g*_*l*_ = 5 × 10^−5^S cm^−2^ for both basal and apical dendrites.

### Numerical methods

Simulations are carried out in MATLAB using the TREES toolbox package [18]. The numerical simulations in Figs 1, 3, S1 and S2 use standard functions described in the toolbox. The non-parametric numerical optimisation in Fig 2 follows an algorithm adapted from an earlier study [9]. The algorithm assigns radii to seven segments of a cable modelled using the TREES toolbox and uses the MATLAB function ‘fminsearch' to maximise the current transfer to the proximal end. This is repeated 50 times to produce a maximum over all trials. The radii of the six distal segments are fitted to a continuous quadratic equation *ax*^2^+*bx*+*c* (as described in [9]) to produce the numerical results of Fig 2.

A function to map an optimal radius profile onto an arbitrary dendritic morphology will be published in the TREES toolbox to accompany this paper.

## Supporting Information

S1 Text Overview of supporting information. Derivation of the arbitrary-radius cable equation. Validity of the multiple scales approximation in real dendrites. Extending the approximation to account for higher-order terms, transients, and branched structures.
(PDF)

S1 Fig Regions of reconstructed dendrites where the asymptotic approximation holds strongly. (**A**) Example reconstructions with regions where *ϵ* > 0.001 highlighted in red. (**B**) Distribution of *ϵ* by cell class for the morphologies described in the online material.
(TIFF)

S2 Fig Second-order approximation provides better results when *ϵ* is larger. Simulated (red), leading-order (black), and second-order (blue) voltage profiles for currents injected at two different points (solid and dashed lines respectively) in the linearly tapering cable (inset).
(JPG)

S3 Fig Asymptotic approximation allows recovery of classical cable properties. (**A**) Time course of voltage at 5, 7.5, and 10ms after current injection at two different sites (solid and dashed lines respectively) on a quadratically tapering cable (inset). (**B**) Steady-state voltage profile in a simple branched structure for current injection at the site with the highest voltage.
(JPG)
