# Supplementary material for: Optimal Current Transfer in Dendrites
Source: PLoS Comput Biol. 2016 May 4;12(5):e1004897. doi: 10.1371/journal.pcbi.1004897 (PMC4856390; doi:10.1371/journal.pcbi.1004897)
Supplement: S1 Text — Derivation of the arbitrary-radius cable equation. Validity of the multiple scales approximation in real dendrites. Extending the approximation to account for higher-order terms, transients, and branched structures. (PDF) [file pcbi.1004897.s001.pdf]

Optimal current transfer in dendrites: Supporting Information

Alex D. Bird[1,2,3] and Hermann Cuntz[4,5]
[1]Warwick Systems Biology Centre, [2]Warwick Systems Biology Doctoral Training Centre, and
[3]School of Life Sciences, University of Warwick, United Kingdom.
[4]Ernst Strüngmann Institute (ESI) for Neuroscience in Cooperation with Max Planck Society
and [5]Frankfurt Institute for Advanced Studies, Frankfurt-am-Main, Germany.

1 Overview

The body of the paper gives the key results and their implications, alongside the derivations of the first-order multiple scales approximation and the optimal taper profile. In this section, we first give a derivation of the cable equation for an arbitrary radius profile. The resulting generalised cable equation is well known, but the derivation is rarely stated. We then discuss the regions of real dendrites where the first-order approximation holds and demonstrate that is a general phenomenon. Finally, we illustrate the utility of the approximation in more general situations: showing how to produce a higher-order approximation, resolve transients and compute the voltage in a complex dendritic structure.

2 Derivation of the cable equation for an arbitrary dendritic radius profile

Consider a section of dendrite with continuously varying radius $r(x)$. Take a section of cable from x to $x+\Delta$ with voltages $V(x)$ and $V(x+\Delta)$ at the respective ends. Then Ohm's Law gives that the current I flowing through this segment obeys

$$V(x+\Delta) - V(x) = -IR \tag{1}$$

where the resistance R is the resistance of the segment given by

$$R = \frac{r_a \Delta^2}{\int_x^{x+\Delta} A_c(s) ds} \tag{2}$$

for r_a the axial resistance and $A_c(x) = \pi r^2(x)$ the cross-sectional area of the dendrite at x. Rearranging the first equation to solve for I gives

$$I = -\frac{\int_x^{x+\Delta} A_c(s) ds}{r_a \Delta} \frac{V(x+\Delta) - V(x)}{\Delta} \tag{3}$$

and taking the limit $\Delta \downarrow 0$ gives the current-voltage relationship

$$I(x) = -\frac{A_c(x)}{r_a} \frac{\partial V}{\partial x} \tag{4}$$

The capacitative and leak currents over the region $(x, x+\Delta)$ obey respectively

$$\begin{aligned} I_C(x) &= C A_s(x, x+\Delta) \frac{\partial V}{\partial t} \\ I_l(x) &= g_l A_s(x, x+\Delta)(V - E_0) \end{aligned} \tag{5}$$

for C the capacitance and g_l the conductance per unit area and

$$A_s(x, x+\Delta) = 2\pi \int_x^{x+\Delta} r(s) \sqrt{1 + (r'(s))^2} ds \tag{6}$$

the surface area of the region. Using conservation of current, we have

$$I_C + I_l = I(x) - I(x+\Delta) \tag{7}$$

So

$$\begin{aligned} C\frac{\partial V}{\partial t} &= g_l(E_0 - V(x)) - \frac{\Delta}{A_s(x, x+\Delta)} \frac{I(x+\Delta) - I(x)}{\Delta} \\ C\frac{\partial V}{\partial t} &= g_l(E_0 - V(x)) - \frac{1}{2\pi r(x)\sqrt{1 + (r'(x))^2}} \frac{\partial I}{\partial x} \end{aligned} \tag{8}$$

where in the second equation we have again taken the limit $\Delta \downarrow 0$. Combining this with the axial current equation Eq (4) (and dividing by the conductance g_l) gives

$$\tau \frac{\partial V}{\partial t} = E_0 - V + \frac{1}{2 r_a g_l r(x)\sqrt{1 + (r'(x))^2}} \frac{\partial}{\partial x}\left[r^2(x) \frac{\partial V}{\partial x} \right] \tag{9}$$

This is the cable equation for an arbitrary radius function $r(x)$. Sealed end boundary conditions with non-negligible terminal area (at $x = L$) imply

$$\left(V + \tau_l \frac{\partial V}{\partial t} + \frac{1}{r_a g_l} \frac{\partial V}{\partial x} \right)\bigg|_{x=L} = 0 \tag{10}$$

At the proximal end, assume that an isopotential soma gives

$$V|_{x=0} = E_0 \tag{11}$$

or we allow the cable to be semi-infinite with voltages decaying as $x \to -\infty$. The cable is initially at rest, with

$$V_{t=0} = E_0 \tag{12}$$

We seek to consider the propagation of a δ current injection at any point x' proximally towards the somatic end.

3 Validity of approximation in real dendrites

We have numerically studied locations where $\epsilon \ll 1$ in reconstructed dendritic morphologies (S1 Fig). Current injection was simulated numerically across the dendritic tree and the highest ratio of voltage change to radius change at each location was determined. The majority of reconstructed dendrites admit very low values of ϵ, although termination points and branch points can have higher values. At branch points the radius can change sharply between a parent and daughter branch, whereas towards the end of dendrites the sealed-end effect can cause shallower voltage attenuation.

4 Extending the approximation

4.1 Higher-order terms

To obtain approximations valid for larger values of ϵ, it is possible to employ a higher-order approximation. We seek solutions of the form

$$w(x) = \mathrm{Re}\{\mu(\epsilon x) e^{i \int^x \sigma(\epsilon s) ds}\} \tag{13}$$

for μ and σ real. Substituting this into Eq 8 in the main text and comparing real and imaginary parts gives

$$\begin{aligned} 2\mu'\sigma + \mu\sigma' &= 0 \\ \epsilon^2 \mu'' - \mu(f + \sigma^2) &= 0 \end{aligned} \tag{14}$$

The first equation gives $\mu^2\sigma = \mathrm{A}$ for some constant A, the second can be solved by expanding μ and σ in powers of ϵ^2 such that $\mu = \mu_0 + \epsilon^2\mu_1$ and $\sigma = \sigma_0 + \epsilon^2\sigma_1$. Similarly, f can be written as $f_0 + \epsilon^2 f_1$ where $f_0 = \frac{2 r_a g_l}{\rho}$ and f_1 makes up the rest. Comparing coefficients of ϵ yields

$$\begin{aligned} \sigma_0 &= i f_0^{\frac{1}{2}} \\ \mu_0 &= \left(\frac{1}{\sqrt{2}} - \frac{i}{\sqrt{2}} \right) \mathrm{A} f_0^{-\frac{1}{4}} \\ \sigma_1 &= \frac{\mu_0'' - \mu_0 f_1}{2\sigma_0\mu_0} \\ \mu_1 &= -\frac{\mu_0\sigma_1}{2\sigma_0} \end{aligned} \tag{15}$$

where we have derived the equations for μ from expanding the $\mu^2\sigma = \mathrm{A}$ equation in each case. A results from matching the boundary conditions as above. The second-order approximation can give a substantially better result when ϵ is not very small, for example where there is sharp taper in a narrow region of dendrite (S2 Fig).

4.2 Transient solution

Consider again the full equation

$$\tau_L \frac{\partial v}{\partial t} = -v + \frac{1}{r_a g_l r(x)\sqrt{1+(r'(x))^2}} \frac{\partial}{\partial x}\left[r^2(x)\frac{\partial v}{\partial x}\right] \tag{16}$$

where $v = V - E_0$ is the voltage above rest. The transform $w = \rho^\epsilon v$ gives

$$\tau_L \frac{\partial w}{\partial t} = -w + \frac{1}{f(\epsilon x)}\frac{\partial^2 w}{\partial x^2} \tag{17}$$

and introducing θ such that $w = \theta e^{-t/\tau_l}$ gives

$$\tau_L \frac{\partial \theta}{\partial t} = \frac{1}{f(\epsilon x)}\frac{\partial^2 \theta}{\partial x^2} \tag{18}$$

Seeking separable solutions of the form $\theta(x,t) = X(x)\,T(t)$ gives the relationship

$$\frac{\tau_L}{T}\frac{\partial T}{\partial t} = \frac{1}{Xf(\epsilon x)}\frac{\partial^2 X}{\partial x^2} = -k^2 \tag{19}$$

for some constant k. Then

$$T_k = e^{-\frac{k^2 t}{\tau_l}} \qquad \text{and} \qquad W_k \approx \sqrt{\frac{\lambda(x)}{ik}}\left[A_k e^{\int^x \frac{ik}{\lambda(s)}ds} + B_k e^{-\int^x \frac{ik}{\lambda(s)}ds}\right] \tag{20}$$

with A_k and B_k determined by the boundary conditions. The voltage profile at different times after current injection into a tapering cable is given in S3a Fig.

4.3 Complex dendritic structures

It is possible to compute the propagation of voltage in more complex dendritic structures. This can be done recursively from the distal points of the dendritic tree. When two child branches with proximal conductances G_{C1} and G_{C2} respectively meet a parent dendrite at $x = L$, the distal conductance of the parent will be $G_E = G_{C1} + G_{C2}$. To proceed, we require the proximal conductance of the parent dendrite G_0. The general voltage solution in the parent dendrite can be written in terms of hyperbolic functions as

$$v(x) = \sqrt{\frac{\lambda(x)}{\lambda(L)}}\left[\cosh\left(\int_x^L \frac{1}{\lambda(s)}ds\right) + \left(\lambda(L)\frac{G_E}{G_{\lambda(L)}} - \frac{\lambda'(L)}{2}\right)\sinh\left(\int_x^L \frac{1}{\lambda(s)}ds\right)\right]v_L \tag{21}$$

where v_L is the distal voltage and $G_{\lambda(L)} = \frac{\pi\rho^2(L)}{r_a}$ is the cross-sectional conductance of the distal end itself. Then the voltage at the proximal end will be v_0

$$v_0 = \sqrt{\frac{\lambda(0)}{\lambda(L)}}\left[\cosh\left(\int_0^L \frac{1}{\lambda(s)}ds\right) + \left(\lambda(L)\frac{G_E}{G_{\lambda(L)}} - \frac{\lambda'(L)}{2}\right)\sinh\left(\int_0^L \frac{1}{\lambda(s)}ds\right)\right]v_L \tag{22}$$

and the current flowing here will be

$$\begin{aligned} I_0 = \frac{v_L G_{\lambda(0)}}{\sqrt{\lambda(L)\lambda(0)}}&\left[\left(\lambda(L)\frac{G_E}{G_{\lambda(L)}} - \frac{\lambda'(L)}{2} + \frac{\lambda'(0)}{2}\right)\cosh\left(\int_0^L \frac{1}{\lambda(s)}ds\right)\right. \\ &\left. + \left(\lambda(L)\lambda'(0)\frac{G_E}{2G_{\lambda(L)}} - \frac{\lambda'(L)\lambda'(0)}{4} + 1\right)\sinh\left(\int_0^L \frac{1}{\lambda(s)}ds\right)\right] \end{aligned} \tag{23}$$

where $G_{\lambda(0)} = \frac{\pi\rho^2(0)}{r_a}$. Then $G_0 = \frac{I_0}{v_0}$, giving

$$G_0 = \frac{G_{\lambda(0)}}{\lambda(0)}\left[\frac{\lambda(L)\frac{G_E}{G_{\lambda(L)}} - \frac{\lambda'(L)}{2} + \frac{\lambda'(0)}{2} + \left(\lambda(L)\lambda'(0)\frac{G_E}{2G_{\lambda(L)}} - \frac{\lambda'(L)\lambda'(0)}{4} + 1\right)\tanh\left(\int_0^L \frac{1}{\lambda(s)}ds\right)}{1 + \left(\lambda(L)\frac{G_E}{G_{\lambda(L)}} - \frac{\lambda'(L)}{2}\right)\tanh\left(\int_0^L \frac{1}{\lambda(s)}ds\right)}\right] \tag{24}$$

This allows analytic determination of the conductance across a complex dendritic structure. To determine the corresponding voltage, consider an injection of current I_{app} at a site on some section at $x = x'$. Along this section, the voltage can be

determined in terms of hyperbolic functions as above, with constants fixed by the current-voltage relationships at each end of the segment and continuity of voltage and conservation of current at the injection site. This gives

$$\begin{aligned} v_{x>x'}(x) &= \sqrt{\lambda(x)}\left[A_2\cosh\left(\int_{x'}^{x}\frac{1}{\lambda(s)}ds\right) + B_2\sinh\left(\int_{x'}^{x}\frac{1}{\lambda(s)}ds\right)\right] \\ v_{x<x'}(x) &= \sqrt{\lambda(x)}\left[A_1\cosh\left(\int_{x}^{x'}\frac{1}{\lambda(s)}ds\right) + B_1\sinh\left(\int_{x}^{x'}\frac{1}{\lambda(s)}ds\right)\right] \end{aligned} \tag{25}$$

with

$$\begin{aligned} A_2 &= \frac{r_a\sqrt{\lambda(x')}}{\pi\rho^2(x')}\left[\frac{1}{\Omega_L-\Omega_0}\right]I_{app} = A_1 \\ B_2 &= \frac{r_a\sqrt{\lambda(x')}}{\pi\rho^2(x')}\left[\frac{\Omega_L}{\Omega_L-\Omega_0}\right]I_{app} \\ B_1 &= \frac{r_a\sqrt{\lambda(x')}}{\pi\rho^2(x')}\left[\frac{\Omega_0}{\Omega_L-\Omega_0}\right]I_{app} \\ \Omega_0 &= \frac{\left(\frac{\lambda'(0)}{2}-\lambda(0)\frac{G_0}{G_{\lambda(0)}}\right)\cosh\left(\int_0^{x'}\frac{1}{\lambda(s)}ds\right)+\sinh\left(\int_0^{x'}\frac{1}{\lambda(s)}ds\right)}{\left(\lambda(0)\frac{G_0}{G_{\lambda(0)}}-\frac{\lambda'(0)}{2}\right)\sinh\left(\int_0^{x'}\frac{1}{\lambda(s)}ds\right)-\cosh\left(\int_0^{x'}\frac{1}{\lambda(s)}ds\right)} \\ \Omega_L &= \frac{\left(\frac{\lambda'(L)}{2}-\lambda(L)\frac{G_E}{G_{\lambda(L)}}\right)\cosh\left(\int_{x'}^{L}\frac{1}{\lambda(s)}ds\right)+\sinh\left(\int_{x'}^{L}\frac{1}{\lambda(s)}ds\right)}{\left(\lambda(L)\frac{G_E}{G_{\lambda(L)}}-\frac{\lambda'(L)}{2}\right)\sinh\left(\int_{x'}^{L}\frac{1}{\lambda(s)}ds\right)-\cosh\left(\int_{x'}^{L}\frac{1}{\lambda(s)}ds\right)} \end{aligned} \tag{26}$$

The voltage attained at the two ends of the section can be used to provide a simple initial condition for voltages in neighbouring dendrites. An example of the voltage profile in a branched morphology is shown in S3b Fig.
