# Supplementary figures and images for: Optimal Current Transfer in Dendrites
Source: PLoS Comput Biol. 2016 May 4;12(5):e1004897. doi: 10.1371/journal.pcbi.1004897 (PMC4856390; doi:10.1371/journal.pcbi.1004897)

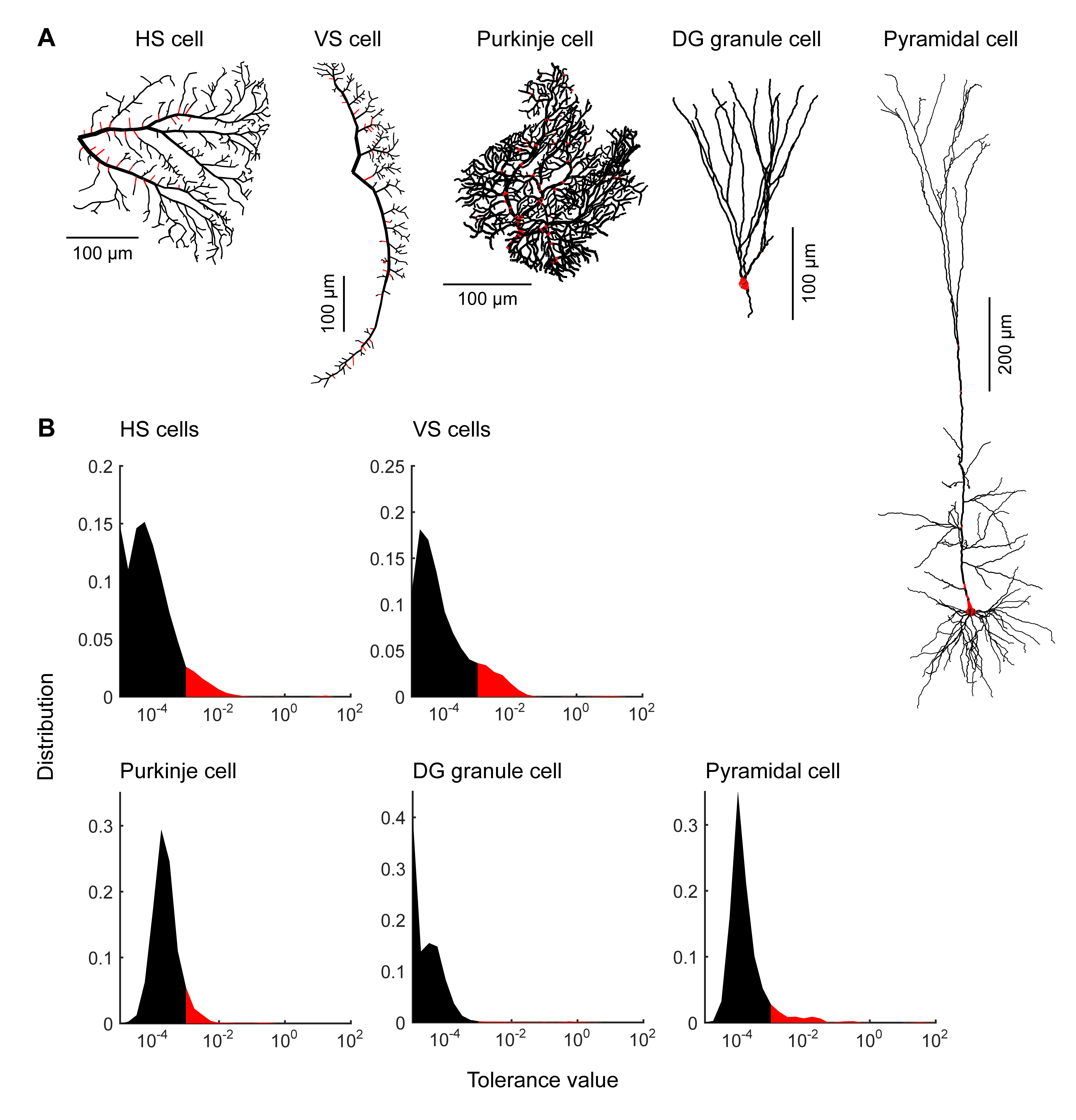

Supplement: S1 Fig — (A) Example reconstructions with regions where ϵ > 0.001 highlighted in red. (B) Distribution of ϵ by cell class for the morphologies described in the online material. (TIFF) [file pcbi.1004897.s002.tiff]

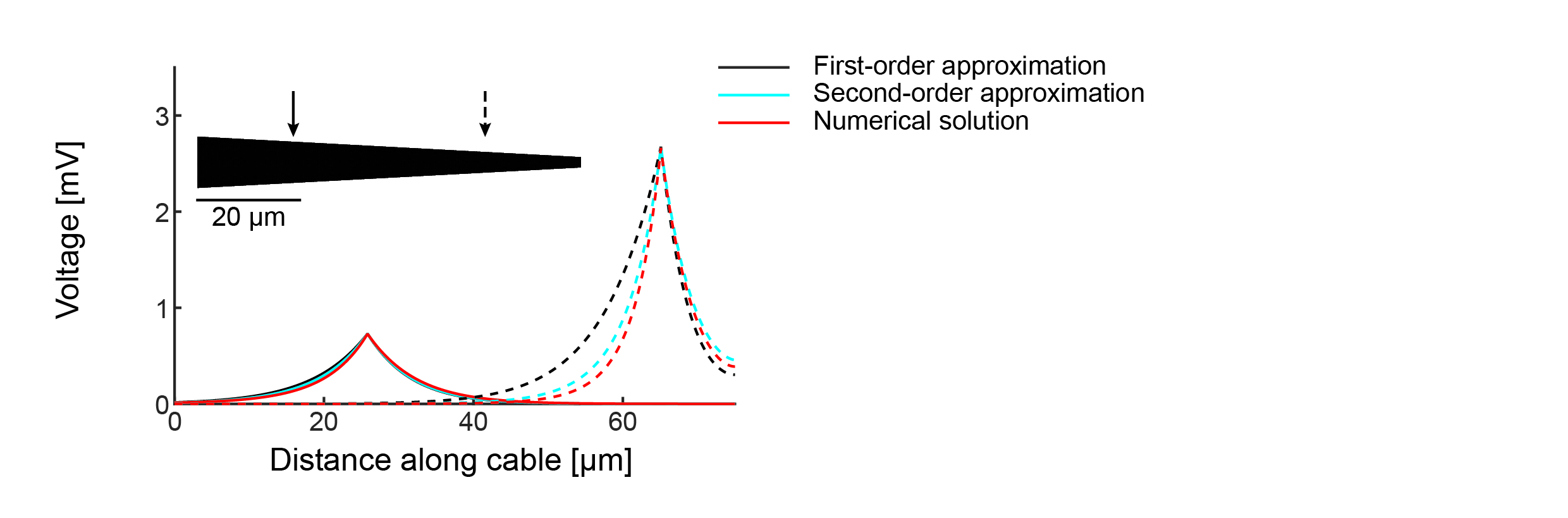

Supplement: S2 Fig — Simulated (red), leading-order (black), and second-order (blue) voltage profiles for currents injected at two different points (solid and dashed lines respectively) in the linearly tapering cable (inset). (JPG) [file pcbi.1004897.s003.jpg]

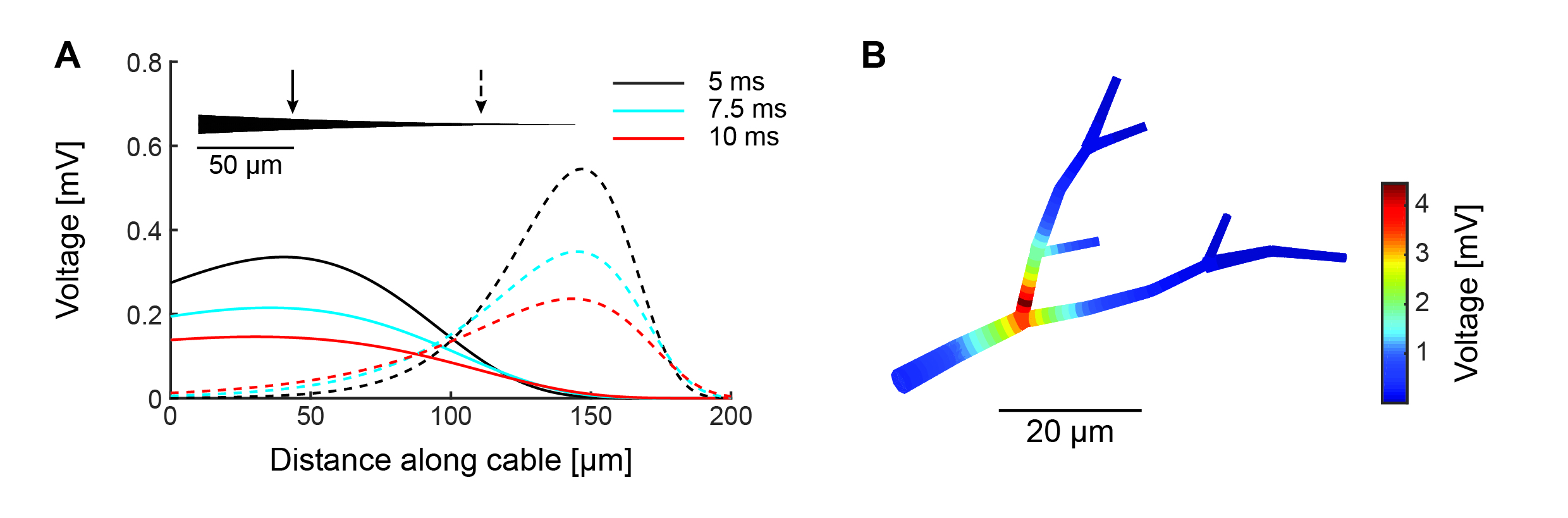

Supplement: S3 Fig — (A) Time course of voltage at 5, 7.5, and 10ms after current injection at two different sites (solid and dashed lines respectively) on a quadratically tapering cable (inset). (B) Steady-state voltage profile in a simple branched structure for current injection at the site with the highest voltage. (JPG) [file pcbi.1004897.s004.jpg]
